# Detection limits of several commercial reverse transcriptase enzymes: impact on the low- and high-abundance transcript levels assessed by quantitative RT-PCR

**DOI:** 10.1186/1471-2199-8-93

**Published:** 2007-10-22

**Authors:** Jean-Philippe Levesque-Sergerie, Mathieu Duquette, Catherine Thibault, Louis Delbecchi, Nathalie Bissonnette

**Affiliations:** 1 Dairy and Swine Research and Development Centre, Agriculture and Agri-Food Canada, P.O.Box 90, STN Lennoxville, Sherbrooke, Quebec, J1M 1Z3, Canada

## Abstract

**Background:**

In functional genomics, transcript measurement is of fundamental importance. Quantitative reverse transcription polymerase chain reaction (qRT-PCR) assays are the most popular technology and depend on the initial molecular step, the reverse transcription (RT). This study provides a complex overview of the influence of elements such as RT systems, amount of background RNA, and transcript abundance on the efficiency of qRT-PCR. Using qRT-PCR, we compared the efficiency of some commonly used RT systems and measured the production of PCR-amplifiable products and the influence of PCR inhibitor contents.

**Results:**

The qRT-PCR assays were conducted using the TaqMan system, although we also tested the SYBR Green I chemistry, which is not compatible with all the RT systems. When dealing with low-abundance transcripts, the SuperScript II system generated more detectable molecules than the four other systems tested: Sensiscript, Omniscript, SuperScript III and PowerScript (*P *< 0.05). However, the Sensiscript and PowerScript systems were more efficient for detecting high-abundance transcripts in the presence of 1 to 2 μg background RNA (*P *< 0.05). The most striking aspect was the influence of the dilution of the RT reaction on the subsequent PCR. Indeed, some inhibition was released when diluted RT reactions were used for the quantitative PCR measurements. Furthermore, the amount of background RNA in the RT reaction was also a major component influencing a downstream step in qRT-PCR, the PCR reaction. Whereas Sensiscript was less biased, the other systems contained an important source of PCR inhibitors, interfering as much as 70% with the qRT-PCR.

**Conclusion:**

This study provides a complex overview of the influence of elements such as RT systems, qRTPCR chemistry, amount of background RNA, and transcript abundance on the efficiency of qRT-PCR. Whereas the most significant influencing factor is the presence of inhibitors in the RT systems, total background RNA is also a major influencing component that affects the PCR reaction. Whenever the aim of a study is to obtain a precise gene expression measurement or to profile the global transcriptome (e.g. microarray), the RT step is critical and should be examined with care.

## Background

In functional genomics, transcript measurement is of fundamental importance, since it not only reveals the activity of a genome but also delivers information on the regulation of biochemical pathways. In that connection, many efforts are directed toward improving detection technologies that hold promise for the discovery of new genes or linking gene activity to new biochemical pathways involved in the development and regulation of mammalian cells. Among the most popular technologies, the microarray tool is used for large-scale gene expression profiling, while the quantitative reverse transcription polymerase chain reaction (qRT-PCR) assay is applied when a precise gene-by-gene interrogation is required. One of the limitations of current technologies, such as microarray, is that a relatively large amount of cDNA is required to generate labelled cDNA [1]. Irrespective of our ability to amplify RNA from tissue, all methods depend on the efficiency of the initial molecular step, the reverse transcription (RT). This is of prime importance when the substrate is limited (nanograms of RNA), such as in biopsies, lasercapture microdissection, historical samples, or specialised cells that are very poor in RNA, such as spermatozoa. Whether the problem is filling the need for micrograms of cDNA in probing microarrays, or depicting an accurate gene expression profile using the qRT-PCR approach, an efficient, sensitive and reliable *RNA-to-cDNA *conversion step is required. Indeed, reverse transcriptase (RTase) needs to be efficient over a wide dynamic range in order to efficiently convert both high-abundance and rare transcripts into cDNA, despite the total amount of "background" RNA in the tube assay. This is one of the most crucial steps in a quantitative study such as qRT-PCR, and it is no less important for whole transcriptome interrogation in microarray studies.

Quantitative RT-PCR is by all means the most accurate approach to building a gene expression profile. Whereas much attention is dedicated to real-time technology for studying the quantitative aspect of transcript abundance, the importance of the RTase contribution is generally a trivial feature. One author has long been raising concerns about the influence of the RT step in qRT-PCR [2,3]. However, no studies have reported a quantitative appreciation of either the influence of the amount of background RNA in the RT assay or the influence of RT conditions on the *RNA-to-cDNA *conversion capacity of low copy number transcripts. This is of prime importance, since transcripts of gene regulators or modulators, such as transcription factors, silencers and enhancers, are often present in low abundance. Indeed, these rare transcripts are sometimes not detected by microarray [4] but rather when a normalisation step such as suppressive subtractive hybridisation is performed, allowing a 10-fold enrichment in lowabundance transcripts [5]. Evaluating the capacity and detection limits of different commercial RT systems in regard to these conditions is fundamental to maximising the sensitivity of the qRT-PCR. For that reason, the first stage of gene expression analysis, the RT step, was examined in order to give further directives about the appropriate choice of RT system for a qRT-PCR assay.

In this study, we addressed the efficiency of different commercial RTases and assessed their sensitivity in their respective systems, i.e. following the vendors' recommendations. We found that, whereas the *RNA-to-cDNA *conversion of abundant transcripts is efficient with all commercial RT systems tested in the presence of various amounts of background RNA, cDNA cannot be detected with certain RT systems when low-abundance transcripts are involved. We found that the RT step is critical for gene analysis and that a judicious choice is required when starting RNA material is limited.

## Results

### Reproducibility of the standard curves

The use of serial dilutions of cDNA (RT reactions) to construct the calibration curves generates PCR amplification efficiency superior to 100% when first points are conserved in the slope [6]. This distortion effect is caused by the influence of some PCR inhibitors present in cDNA, a problem that is attenuated in the subsequent diluted samples and for which normalisation procedures have been suggested [7]. To circumvent these wrongfully derived PCR amplification efficiencies and instead use absolute DNA quantification, DNA fragments (purified amplicons of the target gene) were used as reference in this study. The reference cDNA fragments of the *GNPDA *and *EGFP *genes were cloned into a T/A cloning vector, and their identity was confirmed by sequencing. Purified and quantified PCR products of the *GNPDA *and *EGFP *genes were serially diluted down to the attogram (ag: 10^-18 ^gram) and femtogram (fg: 10^-15 ^gram) range, respectively, and were used as standard templates. Quantitative real-time PCR efficiencies were calculated from the given slopes (in triplicate per plate) and showed high efficiency rates per cycle (1.922 <*E *> 1.976) in the DNA calibration curve model [see Additional file 1]. Cycle threshold (Ct) values for each dilution point of the triplicate standard curves within each plate presented a coefficient of variation (CV) below 1%, indicating a very low technical variability (data not shown). Gathering of the qRT-PCR results for the standard curves obtained from several assays (plates) revealed that the molecular assay was highly reproducible, as judged by the low variation around each of the six dilution points: CVs varied between 1.61% and 3.18% for the respective nine plates totalling 27 *EGFP *calibration curves (table S2a, [see Additional file 1]). A clearly linear relationship existed between the dilution points (log of number of copies) and the average Ct values for both the *GNPDA *(*R*^2 ^= 0.9940 ± 0.0068) and the *EGFP *(*R*^2 ^= 0.9929 ± 0.005) reference genes as measured across assays [see Additional file 1]. The detection assay is therefore reliable and sensitive given the high linearity throughout the study. Whereas the TaqMan assay is highly specific due to the prerequisite of both primers and probe annealing for signal detection, the SYBR Green I system relies on the incorporation of a fluorescent dye into double-stranded DNA. Therefore, in order to confirm the specificity of the measured amplicons, melting curve analysis was systematically performed for all samples analysed with the SYBR Green I system. Products from the standard curves showed no primer-dimers, although there was a single sharp peak with the expected melting point in the dissociation curve analysis, as well as a fragment of expected length (78 bp) in acrylamide gel electrophoresis corresponding to the *GNPDA *gene as confirmed by sequencing (data not shown).

### Determination of the amount of the target transcript for conditions of qRT-PCR measurements of abundant and weakly expressed genes

To address the efficiency of commercial RT systems, we set up a qRT-PCR system that could independently monitor the influence of the amount of background RNA on the respective RT systems and their efficiency based on the transcript abundance. The proposed model used different amounts of background RNA, with a fixed amount of the target gene to be converted into cDNA by different commercial RT systems and measured by qPCR. The commercial RT systems tested were Sensiscript, Omniscript, SuperScript II, SuperScript III, and PowerScript. In order to measure this difference on a large spectrum of background RNA, given that DNA and RNA concentrations are important influencing factors [8-10], the study used total RNA extracted from the same testis tissue (Methods). No bias could therefore be attributed to sample preparation or animal individuality. Furthermore, testes are not a significant source of muscle, fat or blood, all tissues known to generate traces of PCR inhibitors that would interfere with qRT-PCR [11]. In addition, we chose to spike the RT assays with an exogenous transcript, the *EGFP *gene, in order to control the amount of target transcripts. The first assay was performed in order to determine the adequate amounts of *EGFP *mRNA to mimic abundant and weakly expressed gene conditions in the RT reactions. The in vitro transcribed *EGFP *mRNA (Methods) was used to identify the detection limits of each of the commercial RT systems under study. For each RT reaction, 2 μg background RNA was used based on the commercial dynamic range statements for all enzymes, with the exception of the Sensiscript RTase (dynamic range <50 ng), for which the RT assays were conducted with 50 ng background RNA (Table 1). One RT assay was performed for each *EGFP *quantity with the different RT systems. These RT reactions contained either 0 fg (0 copies), 1 fg (2,565 copies), 10 fg (25,650copies), 25 fg (64,138 copies), 1 pg (2.57 × 10^6 ^copies), 5 pg (1.28 × 10^7 ^copies), or 10 pg (2.57 × 10^7 ^copies) *EGFP *mRNA. Preliminary results showed a clear linearity in the range of 10 fg to 10 pg between the amount of input *EGFP *mRNA and the number of cDNA copies detected in the PCR reactions (data not shown). However, when the amount of mRNA was set at 1 fg (approximately 2,500 molecules) or less, there were great differences among the RT systems (data not shown). Indeed, in the presence of 1 fg *EGFP *transcripts, only trace amounts of amplifiable cDNA were detected by qRT-PCR with the Omniscript RT system, whereas substantial copies of *EGFP *cDNA were obtained with the other RTases. Therefore, 2,500 copies (1 fg) was chosen as the relatively small target RNA amount to spike in order to create the low transcript abundance condition. This number of mRNA copies corresponds to 250 molecules of cDNA to be quantified, since 1/10 of the RT reaction (2 μl out of 20) is used for the qPCR. Even if there was still an increase in the detected amount of *EGFP *cDNA molecules with 5 or 10 pg *EGFP *mRNA in comparison with 1 pg, the amount of 1 pg *EGFP *mRNA (2.57 × 10^6 ^copies) was chosen to represent the high transcript level condition, since we did not want to measure the RT efficiencies in a saturated or a biologically non-relevant system. Indeed, more than a 1,000 fold increase in transcript level (1 pg versus 1 fg *EGFP *mRNA) may not be biologically appropriate.

**Table 1:** Dynamic range of template RNA amount for the five commercial RT systems tested.

Commercial name	Type of RT^a^	Dynamic range (ng)
Omniscript	?	50–2,000
PowerScript	MMLV	1,000–5,000
Sensiscript	?	< 50
SuperScript II	MMLV	1–5,000
SuperScript III	MMLV	0.01–5,000

^a^Both the Omniscript and the Sensiscript RTases retain RNase H activity. They are different from the RTases of Moloney murine leukaemia virus (MMLV) or avian myeloblastosis virus (AMV) origin (Sensiscript and Omniscript reverse transcriptase handbook, Qiagen). The Sensiscript RTase is a recombinant heterodimeric enzyme expressed in *E. coli*. TheSuperScript II and SuperScript III RTases are an engineered version of MMLV RTase with reduced RNase H activity and increased thermal stability. The PowerScript RTase is a point mutant of MMLV RTase, which lacks the RNase H enzyme and retains wild-type polymerase activity, thus being able to synthesise longer cDNA fragments [35].

### Comparison of the efficiency of five commercial RT systems measured by qRT-PCR in abundant or weakly expressed gene conditions

**Figure 1:**
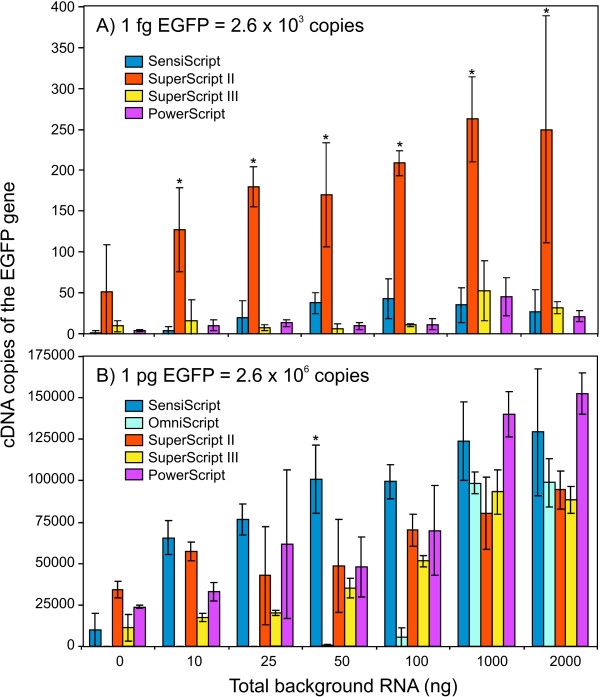
**Quantitative measurements of RT reactions performed with five commercial systems**. Amounts equal to 1/10 (2 μl) of the undiluted RT samples were quantified by qPCR (two-step qRT-PCR). Data are reported as the number of amplifiable cDNA copies in RT reactions spiked with 1 fg (A) or 1 pg (B) *EGFP*-template mRNA. All RT systems were tested and all results are reported, except for the Omniscript system in the presence of low-abundance transcript (A), which was undetectable. All RT reactions were performed and quantified in triplicate for each amount of background RNA ranging from 10 ng to 2 μg. Absolute values of *EGFP *copy number are deduced from a standard curve (Supplementary Table S1a) established with a purified *EGFP *DNA fragment (Methods). Statistical analysis is presented in Supplementary Table S3. *Different from other RT systems within a different background RNA quantity (*P < 0.05*; Supplementary Table S3).

We attempted to evaluate the capacity of different commercial RTases to convert RNA into cDNA in conditions that mimic the detection of an abundant or weakly expressed gene. Notwithstanding the influence of some RT components on the subsequent PCR, we chose first to measure the cDNA copies of the *EGFP *gene in the undiluted RT reaction. Indeed, most of the qRT-PCR studies are carried out using a fraction of the non-purified RT samples, whereas other quantitative assays are performed as "one-tube qRT-PCR" reactions. The comparative study was also performed over a broad range of background RNA in order to measure the influence of background RNA on the RT-PCR yield. Figure 1 presents the number of detectable cDNA copies of the *EGFP *gene measured by qRT-PCR with the five commercial RT systems. It is worthy of note that the performance of the RT systems was influenced by the presence of the background RNA. This influence was apparent for both "low-abundance" (Figure 1A) and "high-abundance" (Figure 1B) conditions. Furthermore, the performance of the RT-PCR systems depended on the gene abundance. When the target gene was present in a low copy number (Figure 1A), more cDNA copies were detected using SuperScript II than the other systems (*P *< 0.05). The Omniscript system was inefficient both for low-abundance transcript conditions (Figure 1A) and, in abundant transcript conditions, when = 100 ng background RNA was used (Figure 1B). In abundant transcript conditions, the different RT systems were more comparable but still presented differences. In the presence of 1 to 2 μg background RNA, both the Sensiscript and the PowerScript systems were superior to Omniscript, SuperScript II and SuperScript III (*P *< 0.05; Table S3). Concerning the influence of the background RNA on the RTase efficiency, the difference measured in the *EGFP *transcript was only significant when comparing RT assays containing amounts of background RNA that differed by more than twofold. For example, results obtained with 50 ng were not different from the 100 ng conditions but statistically different from 1 μg (data not shown). In other words, the impact of the variation of the amount of total RNA on the qRT-PCR is significant only when this variation is pronounced. The Sensiscript system showed a statistically superior yield in the presence of 50 ng background RNA (*P *< 0.05; Figure 1B), according to the specification of the enzyme (Table 1). However, this advantage was only noticeable when the measured transcripts were abundant. The Sensiscript system seemed to be more reliable in the presence of 10 to 100 ng total RNA, as indicated by the weaker standard deviation (SD).

Results reported in Figure 1 are also presented in Table 2 and 3 in terms of copy number and RT-PCR efficiency (detection yield: DY). Ct values are also reported. Since the amount of mRNA molecules used to spike is known, and since the calibration curve is established using defined amounts of molecules, it is possible to calculate the real efficiency of the RT system. The DY of a particular reaction is calculated using the following equation:

**Table 2:** Detection yield, copy number and cycle threshold obtained in qPCR for the RT systems Sensiscript, Omniscript, and SuperScript II.

Background RNA (ng)		0	10	25	50	100	1000	2000
RT systems^a^	Data^b^							
							
**Sensiscript**								
	
	DY (%)	0.62	1.36	7.69	14.57	16.64	13.61	10.63
Low copy	copy num. ± SD	2 ± 2	3 ± 4	20 ± 18	37 ± 11	43 ± 21	35 ± 19	27 ± 24
	(Ct ± SD)	(39.52 ± 0.68)	(39.28 ± 0.71)	(37.19 ± 2.45)	(35.35 ± 0.43)	(35.34 ± 0.72)	(35.61 ± 0.93)	(36.87 ± 2.71)
	
High copy	DY (%)	3.92	25.61	29.79	39.26	38.77	48.33	50.41
	copy num. ± SD	10 068 ± 8880	65 700 ± 9128	76 433 ± 833	100 733 ± 18 034	99 467 ± 9 089	124 000 ± 21 071	129 333 ± 34 020
	(Ct ± SD)	(30.6 ± 7.14)	(24.24 ± 0.21)	(24.02 ± 0.02)	(23.62 ± 0.28)	(23.64 ± 0.14)	(23.29 ± 0.29)	(23.67 ± 0.14)
	
*GNPDA*	copy num. ± SD	0 ± 0	73 ± 25	236 ± 37	691 ± 39	2 150 ± 806	26 395 ± 1 404	42 488 ± 6 724
	(Ct ± SD)	(40 ± 0)	(32 ± 0.62)	(30.03 ± 0.3)	(28.21 ± 0.09)	(26.42 ± 0.58)	(22.22 ± 0.09)	(21.45 ± 0.26)

**Omniscript**								
	
Low copy	DY & copy num.	N/A	N/A	N/A	N/A	N/A	N/A	N/A
	
	DY (%)	0.00	0.00	0.00	0.24	2.16	38.46	38.56
High copy	copy num. ± SD	0 ± 0	0 ± 0	9 ± 15	623 ± 368	5 548 ± 4 152	98 664 ± 5 062	98 927 ± 11 165
	(Ct ± SD)	(40 ± 0)	(40 ± 0)	(38.41 ± 2.75)	(30.73 ± 1.59)	(27.53 ± 2.1)	(22.44 ± 0.08)	(22.44 ± 0.17)
	
*GNPDA*	copy num. ± % (Ct ± SD)	primer dimer	primer dimer	primer dimer	primer dimer	primer dimer	primer dimer	primer dimer

**SuperScript II**								
	
	DY (%)	19.80	49.43	70.14	66.30	81.33	102.44	97.38
Low copy	copy num. ± SD	51 ± 44	127 ± 39	180 ± 18	170 ± 49	209 ± 12	263 ± 40	250 ± 106
	(Ct ± SD)	(34.05 ± 0.04)	(33.41 ± 0.51)	(32.72 ± 0.19)	(32.82 ± 0.42)	(32.47 ± 0.09)	(32.15 ± 0.21)	(32.32 ± 0.7)
	
	DY (%)	13.30	22.33	16.69	18.86	27.31	31.33	36.81
High copy	copy num. ± SD	34 127 ± 4 284	57 277 ± 5 106	42 814 ± 26,175	48 389 ± 24 717	70 062 ± 8 760	80 366 ± 19 132	94 447 ± 10 237
	(Ct ± SD)	(23.52 ± 0.19)	(22.75 ± 0.14)	(23.48 ± 1.19)	(23.18 ± 0.98)	(22.45 ± 0.19)	(22.28 ± 0.4)	(22 ± 0.16)
	
*GNPDA*	copy num. ± SD	5 ± 9	102 ± 48	351 ± 201	1 122 ± 608	2 401 ± 752	19 088 ± 3 540	22 758 ± 1 815
	(Ct ± SD)	(38.79 ± 2.1)	(30.84 ± 1.17)	(28.6 ± 1.12)	(26.43 ± 0.91)	(24.95 ± 0.57)	(21.2 ± 0.35)	(20.86 ± 0.14)

^a^RT systems tested were Sensiscript. Omniscript. SuperScript II. SuperScript III. and PowerScript; low copy. RT samples spiked with 2.6 × 10^3 ^*EGFP *mRNA copies; high copy. RT samples spiked with 2.6 × 10^6 ^*EGFP *mRNA copies.

^b^DY (detection yield) was calculated using the equation described in Results and the copy number (num.) was derived from the calibration curve for a given cycle threshold value (CT) [see Additional file 2];SD. standard deviation.

**Table 3:** Detection yield, copy number and cycle threshold obtained in qPCR for the commercial RT systems SuperScript III and PowerScript

Background RNA (ng)		0	10	25	50	100	1000	2000
RT systems^a^	Data^b^							
							
**SuperScript III**								
	
	DY (%)	3.75	6.30	2.90	2.49	4.07	20.37	12.37
Low copy	copy num. ± SD	10 ± 5	16 ± 20	7 ± 3	6 ± 5	10 ± 1	52 ± 28	32 ± 6
	(Ct ± SD)	(36.61 ± 1.44)	(37.87 ± 1.13)	(36.65 ± 0.6)	(37.19 ± 1.21)	(36.06 ± 0.2)	(33.89 ± 0.98)	(34.41 ± 0.2)
	
	DY (%)	4.48	6.81	7.82	13.78	20.11	36.31	34.48
High copy	copy num. ± SD	11 503 ± 6 190	17479 ± 1 861	20 053 ± 1 168	35 342 ± 4 588	51 592 ± 2 574	93 150 ± 10 206	88 458 ± 6 161
	(Ct ± SD)	(26.28 ± 0.99)	(25.51 ± 0.11)	(25.21 ± 0.09)	(24.37 ± 0.2)	(23.8 ± 0.07)	(22.93 ± 0.16)	(23 ± 0.01)
	
*GNPDA*	copy num. ± SD	0 ± 0	26 ± 3	99 ± 12	142 ± 24	229 ± 31	1 653 ± N/A	5 314 ± 1 090
	(Ct ± SD)	(40 ± 0)	(33.27 ± 0.29)	(30.98 ± 0.16)	(30.35 ± 0.29)	(29.55 ± 0.22)	(26.22 ± N/A)	(24.29 ± 0.37)

**PowerScript**								
	
	DY (%)	1.21	3.97	5.12	3.61	3.61	17.67	8.23
Low copy	copy num. ± SD	3 ± 1	10 ± 5	13 ± 3	9 ± 3	11 ± 5	45 ± 18	21 ± 5
	(Ct ± SD)	(38.34 ± 0.47)	(36.48 ± 0.9)	(35.99 ± 0.41)	(36.56 ± 0.58)	(36.35 ± 0.76)	(34.17 ± 0.75)	(35.27 ± 0.37)
	
	DY (%)	9.29	12.89	23.99	18.69	27.28	54.55	59.43
High copy	copy num. ± SD	23 837 ± 723	33 078 ± 4 291	61 560 ± 340320	47 944 ± 9 523	69 982 ± 20 743	139 950 ± 10 479	152 480 ± 9 523
	(Ct ± SD)	(6.01 ± 0.05)	(25.53 ± 0.2)	(24.72 ± 0.77)	(23.2 ± 0.09)	(24.42 ± 0.45)	(23.33 ± 0.12)	(23.2 ± 0.09)
	
*GNPDA*	copy num. ± SD	2 ± 1	31 ± 3	168 ± 16	35 ± 8	58 ± 40	2 440 ± 650	6 118 ± 308
	(Ct ± SD)	(36.85 ± 1.26)	(32.47 ± 0.18)	(30 ± 0.11)	(32.29 ± 0.35)	(31.9 ± 1.39)	(26.07 ± 0.38)	(24.69 ± 0.07)

^a^RT systems tested were Sensiscript. Omniscript. SuperScript II. SuperScript III. and PowerScript; low copy. RT samples spiked with 2.6 × 10^3 ^*EGFP *mRNA copies; high copy. RT samples spiked with 2.6 × 10^6 ^*EGFP *mRNA copies.

^b^DY (detection yield) was calculated using the equation described in Results and the copy number (num.) was derived from the calibration curve for a given cycle threshold value (CT) [see Additional file 2];SD. standard deviation.

DY=cDNA copies detected by qRT-PCRNumber of EGFP copies spiked in the RT sample×100
 MathType@MTEF@5@5@+=feaafiart1ev1aaatCvAUfKttLearuWrP9MDH5MBPbIqV92AaeXatLxBI9gBaebbnrfifHhDYfgasaacH8akY=wiFfYdH8Gipec8Eeeu0xXdbba9frFj0=OqFfea0dXdd9vqai=hGuQ8kuc9pgc9s8qqaq=dirpe0xb9q8qiLsFr0=vr0=vr0dc8meaabaqaciaacaGaaeqabaqabeGadaaakeaacqqGebarcqqGzbqwcqGH9aqpdaWcaaqaaiabbogaJjabbseaejabb6eaojabbgeabjabbccaGiabbogaJjabb+gaVjabbchaWjabbMgaPjabbwgaLjabbohaZjabbccaGiabbsgaKjabbwgaLjabbsha0jabbwgaLjabbogaJjabbsha0jabbwgaLjabbsgaKjabbccaGiabbkgaIjabbMha5jabbccaGiabbghaXjabbkfasjabbsfaujabb2caTiabbcfaqjabboeadjabbkfasbqaaiabb6eaojabbwha1jabb2gaTjabbkgaIjabbwgaLjabbkhaYjabbccaGiabb+gaVjabbAgaMjabbccaGiabdweafjabdEeahjabdAeagjabdcfaqjabbccaGiabbogaJjabb+gaVjabbchaWjabbMgaPjabbwgaLjabbohaZjabbccaGiabbohaZjabbchaWjabbMgaPjabbUgaRjabbwgaLjabbsgaKjabbccaGiabbMgaPjabb6gaUjabbccaGiabbsha0jabbIgaOjabbwgaLjabbccaGiabbkfasjabbsfaujabbccaGiabbohaZjabbggaHjabb2gaTjabbchaWjabbYgaSjabbwgaLbaacqGHxdaTcqaIXaqmcqaIWaamcqaIWaamaaa@914D@

To assess the DY at a specific dilution point, 1/10 of the spiked amount is used in the calculation, since 2 μl of the 20-μl RT reaction was used in the PCR assay. Consequently, the initial numbers of molecules used for the calculation of the high (1 pg = 2.56 × 10^6 ^molecules) and low (1 fg = 2.56 × 10^3 ^molecules) transcript amounts were, respectively, 2.56 × 10^5 ^and 2.56 × 10^2 ^copies. Calculation of the DY demonstrated once again the inefficiency of the Omniscript system in low-abundance transcript and low background RNA conditions (Table 2). When the detection system was set to measure a low-abundance gene in a 2-μg background RNA assay, SuperScriptII was the only system that seemed to recover almost 100% of the seeded transcripts, whereas the Sensiscript, SuperScript III, and PowerScript systems detected only 10.63%, 12.37%, and 8.23% of the transcripts, respectively. In contrast, PowerScript and Sensiscript were the best-performing systems in the presence of high-abundance transcripts as judged by the DY, which exceeded 50% in 2-μg background RNA assays. The RT systems could therefore be saturated or influenced by inhibitory factors, a dimension that we examine in the next section.

We also verified the efficiency of the RT systems in converting into cDNA a testis transcript, i.e.a transcript present in the background RNA. We quantified the number of copies of *GNPDA *cDNAs in the same RT samples that we used for detecting the 1 pg *EGFP *transcripts (Figure 1B). This time, the RT samples were quantified using the SYBR Green I chemistry (*GNPDA *gene) and the appropriate qRTPCR primers [see Additional file 2]. The results are reported in Table 2 and 3, and the statistical analysis for the *EGFP *and *GNPDA *quantifications are presented in supplementary tables [see Additional files 3 and 4]. The initial evidence showed that no quantification was obtained with the Omniscript system, although the *EGFP *gene was detectable with the TaqMan technology using the same RT samples. The Sensiscript system was the most sensitive detection assay for the *GNPDA *gene in the presence of 1 to 2 μg background RNA, a result that was 50% superior to that of SuperScript II (the next efficient system), even if the Sensiscript dynamic range is < 50 ng (Table 1).

### Influence of the RT components on qPCR and inhibition release by dilution

**Figure 2:**
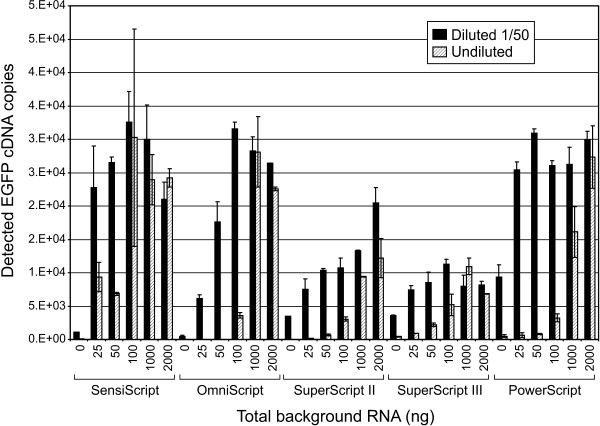
**Effect of RT reaction dilution on the measurements of PCR amplifiable *EGFP *cDNA**. Quantitative PCR was performed in triplicate on undiluted and diluted (1:50) RT samples prepared with each of the five commercial RT systems. The same quantity of *EGFP *mRNA (0.1 pg) was used to spike RT reactions in the presence of different amounts (0 to 2,000 ng) of background RNA.

In the next two experiments, we attempted to evaluate the influence of RT contents and the amount of background RNA on the qRT-PCR assays using the serial dilution method, which is the fastest and most direct way to detect the presence of inhibitors in PCR reactions. The same experimental design was used, i.e. defined conditions of background RNA in the presence of known amounts of target transcript. However, RT reactions were only spiked with 0.1 pg *EGFP *mRNA, since a 1:50 dilution of RT spiked with lower amounts resulted in a loss of signal (data not shown). All RT reactions (one RT per condition) were performed with the same amount of *EGFP *transcripts in the presence of different amounts of background RNA, for the fivecommercial RT systems. Quantitative measurements (in triplicate) of each RT reaction were performed in parallel on undiluted and 1:50 diluted samples (Figure 2). The same quantities of RTase and *EGFP *transcripts were used in the RT reactions. For a given RT system, therefore, when comparing the diluted RT samples together or the undiluted RT samples together, the differences observed can be due only to the influence of the background RNA. In general, more cDNA molecules were detected in the diluted RT samples than in the corresponding undiluted ones. For RT reactions containing 2 μg background RNA, the greatest difference between the diluted and undiluted samples was observed with SuperScript II. In general, the effect of the dilution was weak for the RT performed in the presence of 1 to 2 μg total RNA, whereas this discrepancy increased in the presence of low background RNA, reaching a total absence of detection with undiluted samples at 0 ng background RNA for both the SuperScript II and Omniscript systems. Curiously, when the Omniscript RT assays were diluted, the number of gene copies measured by qRT-PCR for RT containing < 100 ng background RNA was superior to the results for the SuperScript II and III systems and comparable to those for the Sensiscript and PowerScript systems. Indeed, whereas few molecules were detected in undiluted samples in the presence of < 100 ng background RNA, this inhibition was partially released for diluted samples. In other words, even if some RT systems are better than others, using diluted RT samples improved the PCR sensitivity, an effect that was more pronounced in the presence of low background RNA. Indeed, there was a synergetic effect between the amount of background RNA and RT-PCR sensitivity as observed when undiluted samples were quantified, a phenomenon that was attenuated with the dilution. It is not clear how the low background RNA influenced the PCR efficiency in undiluted samples, or how the background RNA could increase the PCR efficiency when diluted along with the RT sample. This aspect is further discussed below with the perspective enunciated by Suslov and Steindler [6], who hypothesised the role of the RTase in the PCR step.

When the diluted samples were quantified, it appeared that almost the same amount of molecules was detected in low versus high background RNA, for at least three of the RT systems. Indeed, the PowerScript, Sensiscript, and SuperScript III systems presented a similar efficiency in the range of 25 to 2,000 ng background RNA. That finding suggested that the difference observed between low and high background RNA, when comparing undiluted RT samples, could not be attributed to weak RTase performance in the presence of low background RNA. Indeed, the poor qRT-PCR detection in the presence of low background RNA was not apparent when the samples were diluted before the qPCR step, indicating that the RT reaction was still efficient in the presence of low background RNA and that the reason for low detection with undiluted samples came after the RTase step. Therefore, it suggests not only that some RT contents influenced subsequent PCR but that the extent of this influence depended on the RT system.

### Quantitative PCR measurement of inhibition by RT components

At the time of writing the present manuscript, an interesting paper reporting quantitative data about the effect of some components of the SuperScript III reaction was published. The authors of that paper reported that the SuperScript III system contains PCR inhibitors and could lead to an overestimation of amplification efficiency under certain conditions [6]. In order to evaluate the importance of this phenomenon with the different commercial RT systems, we followed the same procedure that had already been carried out. We tested the different RT systems in triplicate spiked with 1 pg *EGFP *mRNA, and ran all the RT reactions in triplicate using undiluted and serially diluted (2-, 8-, 16-, 32-, 64-, and 128-fold) samples. The dilution solution contained 50 ng/μl yeast tRNA, and all samples were set to contain a final amount of 2μg background RNA [6]. Again, the number of copies detected was fairly similar within the triplicates, as judged by the low SD (data not shown). The onset of fluorescence (Ct) varied when either the undiluted or the diluted cDNAs were used. However, this gap was variable depending on the RT system used (data not shown). The respective PCR efficiencies were calculated using the equation *E *= 10^(-1/slope) ^and are presented in Table 4 as percentages. These efficiencies were calculated for a slope determined (i) with all points, (ii) without the first point (undiluted RT sample), or (iii) without the first two points (undiluted and two-fold diluted samples) [6]. An efficiency of 100% corresponds to a slope value of -3.32. From these results, we deduced the inhibition percentage (IP), which is calculated using the following equation:

**Table 4:** Inhibition effect of the RT contents of five commercial RT systems measured by qRT-PCR.

**RT systems**	**Slope**^a^	**SD**	**Efficiency**^a, b^		***R^2^***	**SD**	**Average IP**^a^
**Sensiscript**							
*E/R*^2 ^- all points							14.7 ± 6.1%
	-3.393	± 0.030	97.13	± 5.00%	0.9981	± 0.0016	
*E/R*^2 ^- w/o 1st point							
	-3.464	± 0.041	94.42	± 1.53%	0.9983	± 0.0017	
*E/R*^2 ^- w/o 1st 2 points							
	-3.519	± 0.089	92.45	± 3.12%	0.9968	± 0.0029	

**Omniscript**							
*E/R*^2 ^- all points							31.2 ± 23.5%
	-3.227	± 0.054	104.14	± 2.42%	0.9958	± 0.0035	
*E/R*^2 ^- w/o 1st point							
	-3.323	± 0.057	99.98	± 2.37%	0.9957	± 0.0038	
*E/R*^2 ^- w/o 1st 2 points							
	-3.473	± 0.208	94.50	± 8.10%	0.9969	± 0.0019	

**SuperScript II**							
*E/R*^2 ^- all points							59.7 ± 6.8%
	-2.862	± 0.109	123.79	± 6.95%	0.9907	± 0.0029	
*E/R*^2 ^- w/o 1st point							
	-3.004	± 0.122	115.50	± 6.93%	0.9920	± 0.0013	
*E/R*^2 ^- w/o 1–2 points							
	-3.303	± 0.121	100.99	± 5.26%	0.9971	± 0.0034	

**SuperScript III**							
*E/R*^2 ^- all points							79.3 ± 2.9%
	-2.400	± 0.095	161.44	± 10.16%	0.9830	± 0.0057	
*E/R*^2 ^- w/o 1st point							
	-2.583	± 0.083	144.07	± 6.84%	0.9898	± 0.0040	
*E/R*^2 ^- w/o 1–2 points							
	-2.882	± 0.069	122.43	± 4.34%	0.9980	± 0.0024	

**PowerScript**							
*E/R*^2^- all points							69.3 ± 2.1%
	-2.568	± 0.063	145.27	± 5.49%	0.9693	± 0.0113	
*E/R*^2 ^- w/o 1st point							
	-2.759	± 0.168	131.12	± 12.38%	0.9694	± 0.0038	
*E/R*^2 ^- w/o 1–2 points							
	-3.203	± 0.141	105.50	± 6.71%	0.9817	± 0.0167	

^a^Results report the means of triplicate assays for each RT system.

^b^Amplification efficiency is calculated using the equation *E*% = (1 - 10^(-1/slope)^) × 100, where 100% PCR efficiency corresponds to a slope (Δ Ct) of -3.3; *E *and coefficient of correlation (*R*^2^) are derived from the slope of a sample's calibration curve; SD, standard deviation.

IP=Δ cDNA copies of the EGFP reference geneNumber of copies in the 128-fold diluted samples×128×100
 MathType@MTEF@5@5@+=feaafiart1ev1aaatCvAUfKttLearuWrP9MDH5MBPbIqV92AaeXatLxBI9gBaebbnrfifHhDYfgasaacH8akY=wiFfYdH8Gipec8Eeeu0xXdbba9frFj0=OqFfea0dXdd9vqai=hGuQ8kuc9pgc9s8qqaq=dirpe0xb9q8qiLsFr0=vr0=vr0dc8meaabaqaciaacaGaaeqabaqabeGadaaakeaacqqGjbqscqqGqbaucqGH9aqpdaWcaaqaaiabfs5aejabbccaGiabbogaJjabbseaejabb6eaojabbgeabjabbccaGiabbogaJjabb+gaVjabbchaWjabbMgaPjabbwgaLjabbohaZjabbccaGiabb+gaVjabbAgaMjabbccaGiabbsha0jabbIgaOjabbwgaLjabbccaGiabdweafjabdEeahjabdAeagjabdcfaqjabbccaGiabbkhaYjabbwgaLjabbAgaMjabbwgaLjabbkhaYjabbwgaLjabb6gaUjabbogaJjabbwgaLjabbccaGiabbEgaNjabbwgaLjabb6gaUjabbwgaLbqaaiabb6eaojabbwha1jabb2gaTjabbkgaIjabbwgaLjabbkhaYjabbccaGiabb+gaVjabbAgaMjabbccaGiabbogaJjabb+gaVjabbchaWjabbMgaPjabbwgaLjabbohaZjabbccaGiabbMgaPjabb6gaUjabbccaGiabbsha0jabbIgaOjabbwgaLjabbccaGiabigdaXiabikdaYiabiIda4iabb2caTiabbAgaMjabb+gaVjabbYgaSjabbsgaKjabbccaGiabbsgaKjabbMgaPjabbYgaSjabbwha1jabbsha0jabbwgaLjabbsgaKjabbccaGiabbohaZjabbggaHjabb2gaTjabbchaWjabbYgaSjabbwgaLjabbohaZjabgEna0kabigdaXiabikdaYiabiIda4aaacqGHxdaTcqaIXaqmcqaIWaamcqaIWaamaaa@A58A@

where *Δ cDNA copies *is the difference between the number of copies detected in the undiluted RT and the number of copies corresponding to the 128-fold diluted RT sample corrected for the dilution (× 128). The Sensiscript RT system contained the least inhibitory components with only 14.73 ± 6.09% PCR inhibition, followed by the Omniscript RT system (31.23 ± 23.50%). These RT systems are quite reliable, since the estimated amplification efficiency calculated with or without the first two points was close to 100%, which is representative of an unbiased PCR reaction. These results are also in accordance with those reported in Figure 2 for 2 μg background RNA. The worst deviations were observed with the SuperScript III RT system, with an average IP score of 79.31 ± 2.85%. Quite logically, the RT systems providing the highest IPs were also those for which the distortions in amplification efficiencies were the greatest, especially when the first two points were included. This suggested that the higher the starting IP at the first point, the lower the possibility of detecting the correct amplification efficiency. In other words, the most concentrated points are under the biggest inhibitory influence; these points should always be excluded from a calibration curve. In consequence, the same reasoning must be applied to the RT samples; they should not be quantified as undiluted samples but rather in conditions (i.e.at least a 1:10 dilution or following the purification step) that ensure that the subsequent qRTPCR will not be affected by the PCR inhibition effect.

## Discussion

### Dynamic range and detection efficiency of the RT systems

Notwithstanding the ability to detect a broad range of gene copies, the most challenging aspect of a qRT-PCR study is the extraction of the absolute or most representative transcript quantity of an RT sample. It is usually accepted that the RNA quantity of an RT assay determines the choice of RTase based on the manufacturer's specifications. However, it is not clear whether the suggested dynamic ranges take into account the abundance of targeted transcripts. Furthermore, the manufacturer's specifications do not indicate if the RT system is directly transposable to qPCR assays. Depending on the RT system used, we had previously observed inconsistencies between end-point PCR and qPCR measurements (data not shown). We therefore hypothesised that each RT system would have its respective enzyme efficiency and PCR adaptability. Because qPCR assay is more precise–qRT-PCR displays a dynamic range that is three to four times greater than end-point PCR [12]–the qPCR technology is the more suitable approach for such a comparison [13]. Indeed, we were able to detect a five-log range of gene copies [see Additional file 1] using both the TaqMan and SYBR Green I systems.

We established an informative qRT-PCR assay that allowed quantification of an exogenous transcript (*EGFP*) introduced into the RT reactions within defined conditions, with the purpose of testing the influence of the following parameters on PCR reactions: the RT system, the abundance of the quantified mRNA (transcript level), the amount of background RNA, and the dilution. Therefore, the same template was quantified in different conditions, and the differences observed could not be attributed to the nature of the transcript. Then, the RTase was primed with an oligo(dT)_12–18_. Hence, the present paper reports a representative evaluation of the PCRdetectable amount of *mRNA-to-cDNA *copies. In theory, the "random" approach primes the RT at multiple origins along every RNA template, hence producing more than one cDNA molecule per original mRNA target. Nonetheless, we did not observe a better yield in *EGFP *detection with the random priming kit (i.e. the High-Capacity cDNA Archive Kit from Applied Biosystems) when compared to the SuperScript II system, using either diluted or undiluted RT reactions, and the TaqMan or SYBR Green detection system (data not shown). It is well accepted that qPCR is robust and predictable in defined conditions. Indeed, when the quantity of background RNA was constant, and for most of the tested range of mRNA quantity introduced in the RT reactions, we observed a linear relationship between the gene copies detected by qRTPCR and the starting amounts. The amounts of *EGFP *mRNA ranging from 2.2 × 10^4 ^to 2.6 × 10^7 ^copies delivered proportional PCR-detectable cDNA molecules (data not shown). This representation is generally supported in the technical bulletin provided by each company. However, a discrepancy between RT systems was observed at a very low transcript level (1 fg, equivalent to 2,600 molecules of *EGFP *mRNA in the RT assay). We then designed the experiment to statistically explore this difference further by repeating the RT assays using this low amount of transcripts (2.6 × 10^3 ^molecules), as well as 1 pg mRNA (2.6 × 10^6 ^copies; highabundance condition), in the presence of variable amounts of background RNA. Using qPCR to measure the capacity of the RT systems enabled us to determine two types of influence: the transcript abundance (low vs. high) and the amount of background RNA (Figure 1). According to the commercial RT systems and our own results, it is clear that both parameters influence the DY (Table 2 and 3). A significant difference was associated with the abundance of the gene: greater differences between RT systems were observed in low-abundance gene conditions. All quantitative results obtained with undiluted RT samples presented RT systems as being barely efficient for converting low-copy transcripts, with the exception of SuperScript II. Indeed, the SuperScript II system was more sensitive and superior when compared with the other RT systems (Figure 1A). However, this particular aspect is not necessary suitable in most qRT-PCR studies, since the objective is usually directed towards the detection of differences in transcript levels. In other words, "boosting" the low-abundance genes might mask differences between samples.

In a paper reviewing the problems and limitations of qRT-PCR, Bustin reported that Sensiscript and SuperScript II deliver similar signals (Ct values) when detecting high-abundance genes, whereas a fivefold increase was observed with SuperScript II when a medium to low transcript amount was assayed [2]. These observations are in keeping with our findings for lowabundance cDNA copies, although the efficiency of SuperScript II was about 10 times that of Sensiscript. For high-abundance mRNA conditions, however, Sensiscript was superior to SuperScript II. Indeed, when measuring an abundant transcript in the presence of 1 to 2 μg background RNA, Sensiscript was superior to the other tested systems for both the *EGFP *[see Additional file 3] and *GNPDA *[see Additional file 4] genes. Furthermore, Sensiscript was slightly more sensitive in the presence of less RNA and was superior to the other tested systems at 50 ng background RNA (Figure 1B, [see Additional file 3]). Therefore, in spite of the dynamic range of this RT system (< 50 ng/μl; Table 1), the Sensiscript was highly efficient within the 25 to 2,000 ng range.

### Influence of the background RNA

**Figure 3:**
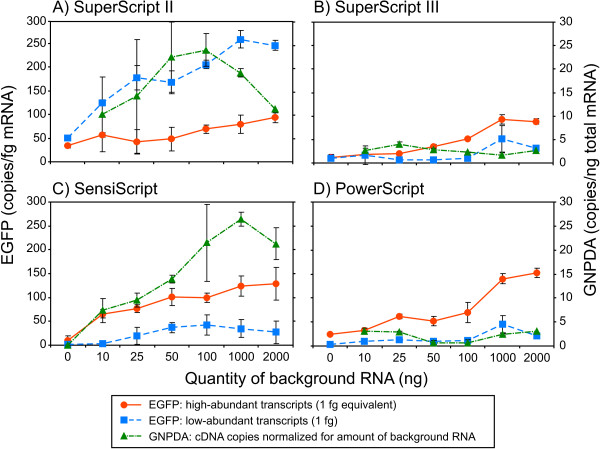
**Influence of the background RNA the on detection of *EGFP *and *GNPDA *cDNAs**. The high-abundance *EGFP *results were reported as 1 fg equivalent (i.e. divided by 1,000), whereas low-abundance *EGFP *results were uncorrected. Quantities of cDNA copies detected for *GNPDA *in total RNA (Table 2 and 3) were corrected for the amount of background RNA. Data for the SuperScript II, SuperScript III, Sensiscript, and PowerScript systems (panels A, B, C, and D, respectively) were plotted using the same scale for comparison purposes.

In their specifications, most of the commercial manufacturers indicate the dynamic range of their systems. However, the manufacturers do not present absolute comparisons of the actual efficiency of their system in terms of the *RNA-to-cDNA *conversion capacity of abundant or rare transcripts in the presence of low background RNA. In the present study, we measured the effects of non-targeted background, i.e. total, RNA on the efficiency of the qRT-PCR, as well as the impact on the PCR step. This effect was measured using an exogenous transcript, *EGFP*, and the endogenous *GNPDA *transcript, i.e. a biological reference present in the background RNA. When detecting the *GNPDA *transcript at 2 μg total RNA, Sensiscript was two times better than the other three RT systems [see Additional file 4], a finding that reflects a high-abundance profile. The average number of *GNPDA *mRNA copies detected with Sensiscript was 42,488 ± 16% in 2 μg total testis RNA (Table 2), which might represent around 430,000 copies in the whole RT sample (1/10 of the RT reaction was used for qPCR assays). When comparing this number to the *EGFP *low and high copy number conditions (2.6 × 10^3 ^and 2.6 × 10^6^molecules, respectively), we may consider this *GNPDA *transcript to be mediumabundance. In order to present an overall picture of these findings, we summarised the three experiments, i.e. the low- and high-abundance *EGFP *and the medium-abundance *GNPDA *transcripts, in Figure 3 (same scales for *EGFP *and *GNPDA *detection). The quantitative data for 1 pg *EGFP *were therefore divided by 1,000 (based on the 1 fg assays), and the *GNPDA *data were corrected for the amount of background RNA in the assays. There is a clear trend toward promoting the detection of high-abundance over medium- and low-abundance transcripts in the presence of low background RNA for all systems except for SuperScript II, which was more efficient with lowabundance transcripts (Figure 3A). Another interesting finding concerns the efficiency of the Sensiscript system (Figure 3C). Although the specificity of the RT was < 50 ng total RNA (Table 1), this system was more efficient with 1 to 2 μg total RNA for medium- and highabundance transcripts. The third aspect that is worthy of note is the effect of background RNA on the endogenous *GNPDA *transcripts. Notwithstanding SuperScript III and PowerScript (Figure 3B and 3D), for which PCR-detected cDNA copies were too low to be appreciated, more molecules were detected with the Sensiscript system when the amount of total RNA increased. This observation is in keeping with the RNA-dependent RTase activity that was previously proposed [8]. However, we observed a saturation effect above 1 to 2 μg background RNA that was more obvious with the SuperScript II RT system and that could not be explained by the dynamic range of the system. Indeed, we also tested the RT systems by increasing the background RNA condition up to 5 μg total RNA. When RT reactions are not diluted, there is no detection improvement from 1 μg up to 5 μg for all RT systems (data not shown). There is therefore no benefit to using more than 1 μg total RNA when undiluted RT reactions are used in qPCR assays.

It is still unclear how an increased amount of background RNA (non-target RNA) introduced in RT reactions increases the PCR sensitivity, a downstream application of the RT step. In this study, we were able to observe a significant effect of the low background RNA when samples were diluted. The dilution effect was partially released when the amount of total RNA was higher (i.e. 1 to 2 μg; Figure 2), but this inhibition release cannot be accounted for by an increased amount of template, since the *EGFP *transcript was not part of the background RNA but rather a constant of the RT reaction. Indeed, variations in qRT-PCR efficiency could not be accounted for by differences in RT preparations either, since the same mix was distributed into reaction tubes containing variable amounts of total RNA, for each RT system tested. Therefore, it became obvious that the amount of background RNA alone accounted for this discrepancy. Furthermore, the phenomenon was only observable when using total RNA. As reported else where [14], we did not observe any RT-PCR performance improvement with tRNA (data not shown).

It has been suggested that RTase may exhibit several activities, including (i)RNAdependent DNA polymerase activity (RT activity) [15,16] (ii) DNA-dependent DNA polymerase activity [17-19] (iii) terminal nucleotidyl transferase-like activity (TdT) [20,21], (iv) strand transfer and displacement ability [22,23], (v) positive effect on PCR activity [6], (vi)DNA binding capacity [6], and (vii) RNase H activity. Whereas both the Omniscript and Sensiscript RTases retain RNase H activity, the SuperScript II and III systems have a mutated RTase with reduced RNase H activity, and PowerScript lacks the RNase H enzyme (Table 1). The results observed in this study may not be attributable to the RNase H activity. Whereas some authors observed that SuperScript III RTase (3.2 to 51.2 U [units]) could have a positive effect on qPCR when introduced in the PCR step [6], others reported the possible loss of PCR sensitivity due to the interaction between the RTase and DNA. One study concluded that this loss of PCR sensitivity is mediated through a specific interaction of the RTase with the primertemplate duplex rather than with *Taq *polymerase itself [14]. Although it was demonstrated that the introduction of nonhomologous RNA relieves the amplification inhibition for the AMV H^- ^RT [24], no such effect was observable with the MMLV H^- ^RT [6,14]. However, the first statement resulted from an experiment using yeast tRNA. It was reported that the amount of total RNA does not affect the inhibitory processes, but this statement was made in regard to an experiment where the proportion of the target gene versus total RNA was maintained in the different SuperScript III RT assays (i.e. by measuring an internal transcript using either 50 ng or 1 μg RNA in the RT assay, the proportion of target gene within the RNA pool is conserved) [6]. In our TaqMan assays, the effect of the background RNA was observed under a constant *EGFP *transcript level, and the influence on the detection can only be mediated by nonhomologous RNA.

Since the amount of target transcripts was equally distributed throughout the RT reactions, and since the same RT content was run as diluted or undiluted samples as described above, an interaction of the background RNA in the post-RT step, i.e. the PCR, was clearly indicated. It is intriguing that the loss of PCR sensitivity was noticeable when the target cDNA was favourably available for the PCR reaction, i.e. in low background RNA conditions where the proportion of the target transcript (*EGPF*) relative to total RNA was high. If the background RNA acted simply as a chelator of PCR inhibitors, it is difficult to explain why tRNA did not have an effect, unless this effect is specific to mRNA. To add a degree of complexity to this interpretation, the loss of PCR sensitivity was partially released when RT samples were diluted. Therefore, we cannot exclude that this variation (difference between diluted and undiluted samples) originated from direct competition between the *Taq *polymerase and the RTase.

### Inhibition by the RT contents

The most striking aspect of our findings is the influence of the RT contents on the subsequent PCR efficiency. This observation was particularly evident for the Omniscript system, where no detection was observed for the *GNPDA *gene, whereas quantification of the *EGFP *gene was possible. Even though three sets of primers were designed and tested, the presence of primer-dimers always masked any relevant quantification. Since the observation was restricted to the Omniscript system, i.e. since no such primer-dimers were detected with the standard curve or any of the other RT systems, it also suggests that the Omniscript system would not be compatible with the SYBR Green I chemistry, although we did not test another testis gene. However, this observation does not rule out the possibility that the presence of inhibitors could interfere with the qRT-PCR reaction by generating the detected primer-dimers. Indeed, we observed a great discrepancy between the undiluted and diluted RT samples. The RT systems produced very different DYs, ranging from no detection with Omniscript to 102% efficiency with SuperScript II in conditions of low-abundance transcripts, which was reduced to 31% in highabundance conditions (Table 2). Furthermore, this profile was greatly distorted in a sidebyside comparison of the undiluted and diluted samples (Figure 2). In some cases, such as with the Omniscript system, a complete absence of PCR-detectable cDNA copies with undiluted samples was observed when more than 10,000 molecules could be detected in 50fold diluted samples. It became evident that some RT systems contain substantial amounts of PCR inhibitors. Consequently, the next experiment was aimed at verifying the extent of this influence on subsequent qPCR evaluations by comparing serial dilutions of RT reactions. Previous studies reported that, when a calibration curve for qPCR is established with RT samples (i.e. serial dilutions), the result is often a PCR efficiency greater than 100% [6,7]. The contribution of some RT components was evident given the observed variations in the slope of the curve, whether it included all dilutions or excluded the first (undiluted sample) or first two dilution points (Table 4). When the slope is inferior to -3.32, the PCR efficiency is superior to 2(10^(-1/slope)^), meaning that each cycle generates more than twice the amount of cDNA copies, which is impossible. This suggests the presence in the RT reaction of PCR inhibitors, which are reduced in diluted samples. All the RT systems tested in the present study, except Sensiscript, contain a substantial amount of inhibitors, given the respective efficiencies that exceed 100%: 104.14 ± 2.42% for Omniscript, 123.79 ± 6.95% for SuperScript II, 161.44 ± 10.16% for SuperScript III, and 145.27 ± 5.49% for PowerScript (Table 4). Using extreme points (undiluted and 128-fold diluted samples), we were able to calculate the average IP. The most inhibitory RT system was SuperScript III (79.31 ± 2.85%), whereas Sensiscript was the least inhibitory with an IP of 14.73 ± 6.09%. Sensiscript was also the RT system that had the highest reliability for amounts of background RNA between 0.1 and 2 μg (Figure 2). While Omniscript was the least sensitive system for both low and abundant target transcripts (Figure 1 and Table 2), it produced a weak IP of 31.23 ± 23.50% (Table 1). However, we observed a major contribution of the primer-dimer occurrence with the Omniscript system when using the SYBR Green I chemistry. The lack of sensitivity attributed to PCR inhibitors was noted before, but the primer-dimer problem was not pointed out [25]. However, the enhanced production of artefacts, presumably primer-dimers, was reported elsewhere [26]. It was also reported that a limited amount of template cDNA or an increased cycle number beyond the linear PCR range causes increased primer-dimer formation [13]. This was not the case here, since this phenomenon was detected with a medium level of *GNPDA *transcripts and in the exponential phase of the reaction. Therefore, the problem of primer-dimer occurrence with Omniscript is inherent to this RT system. To ensure that the overall performance of each RT system was not batch-dependent, we contacted the respective companies. They did not report problems with the batches used for the study. Clearly, all RT systems have their respective attributes (primer-dimer formation and PCR inhibition potential) and, obviously, different RT systems are definitely not compatible within the same qRT-PCR study. Furthermore, samples harbouring different dilutions cannot be compared. Indeed, there is no point in measuring differences between samples if these differences are simply due to a differential concentration of inhibitors or PCR boosters that belong to the RT step. Generally, the experimental design of a qRT-PCR study is established using equal amounts of RNA (total RNA or mRNA). Furthermore, there is a clear trend toward using relative quantification, i.e. to refer to a housekeeping or stable gene, when variations in target gene expression are examined. Indeed, the effect of the RT content (diluted or not) on qPCR should not be affected by the presence of a constant amount of background RNA. However, we cannot exclude that a burst of RT of lowabundance transcripts could have a significant effect on the profile.

Notwithstanding the accuracy of their experimental designs, most of the qRTPCR studies do not consider the deleterious effects of the RT buffer components. In the best situations, RT cleanup using a silica-based column or organic extraction, as suggested by Suslov and Steindler [6], would be performed before RT samples are introduced into the qPCR assay. These conditions are often overlooked for practical reasons: the cleanup step is expensive, organic extraction cannot be applied to large-scale studies, and there is sometimes not enough RNA to allow for the cleanup step–for the systems tested in the present study, more than 50% of the RNA was lost when the samples were passed through the column (data not shown). Indeed, decreased sensitivity [2] and a one-log reduction in the signal [27] when using a spin column for RNA isolation have been reported. Consequently, it might be wise to dilute the RT reactions in order to minimise the inhibition effect [24] and relieve the *Taq *polymerase/RTase competition or interaction [6,14,26], as long as the transcripts are still detectable following the dilution. A 10-fold dilution is required to relieve the inhibition effect by at least 50% [6,25], which would increase the Ct of samples by at least 3.3 units, keeping in mind that a Δ Ct > 5 units is required to declare samples statistically different from no-template controls [28].

### Weak RTase efficiency and reproducibility, or the "Monte Carlo" effect

The RTase enzymes in the PowerScript, SuperScript II and SuperScript III systems are engineered versions of the enzyme derived from the Moloney murine leukaemia virus (MMLV; manufacturers' manuals). The Omniscript and Sensiscript RT enzymes are different from the MMLV or avian myeloblastosis virus (AMV) RTases, and both enzymes retain RNase H activity (Sensiscript and Omniscript reverse transcriptase handbook, Qiagen). Previous work reported that the Omniscript RTase is superior to AMV or MMLV RTases [29]. However, we did not observe such superiority in the Omniscript system under the same conditions (1 to 2 μg RNA). Furthermore, whereas the Omniscript and Sensiscript RTases are the same enzyme, their respective systems do not have similar performances. The nature of the RT enzyme cannot account for the observed discrepancies.

The reproducibility of the RT assays is generally high, as shown by the low SDs of the RT systems tested. However, we observed an inherent limitation in PCR amplification from small amounts of transcripts (1 fg *EGFP*). This observation has been related to the "Monte Carlo" phenomenon [30], which is created by small and random differences in amplification efficiency between individual templates in a population where every template has a different probability of being processed. In other terms, below a certain molar threshold, a specific transcript present in a complex mixture cannot be reproducibly detected [30] and quantified [3,28] Karrer et al. [30] asserted that rare mRNAs (< 0.04% of polyadenylated mRNA) exhibited significant variations in gene detection caused by the "Monte Carlo" effect and that an evaluation would not be quantitative but rather qualitative under these conditions. In our design, 1 fg *EGFP *mRNA represented about 0.00025% of mRNA content in the 10-ng RT sample (taking into account that total RNA contains 3 to 5% mRNA), and 100 to 200 times less in the presence of more background (1 to 2 μg). Therefore, the quantity used could have been subjected to the "Monte Carlo" effect. However, there are several pieces of evidence against this effect. The difference between high and low gene abundance was exacerbated in conditions of low background RNA, a condition where the *EGFP *transcripts were proportionally more abundant. Furthermore, both RT and PCR amplification of *EGFP *and *GNPDA *transcripts, although very low with the PowerScript and SuperScript III systems, were still reproducible and consistent. The PCR step was not influenced by the "Monte Carlo" effect, as demonstrated in the experiment using diluted RT samples: the absence of amplification of cDNAs was partially relieved by dilution. The decreased performance could therefore not be attributed to a weak probability of the cDNA molecule being amplified ("Monte Carlo" effect) but rather to PCR inhibition. Consequently, it appears that the major influencing component is the PCR inhibitors' content itself.

### More robust and qPCR-transposable RT systems are required

Great divergences in efficiency were observed among the commercial RT systems tested in this study, and important IP contributions to qPCR were detected. Both the background RNA and the RT contents are undeniably influencing factors. Therefore, these aspects should be considered with caution. Companies should provide more detailed information on their products, and an RT buffer with information about its impact on PCR should be included in order for reliable qRT-PCR studies to be performed.

Although the influence of RT on quantitative studies has been underlined in published reviews [3,28], no studies have quantified the efficiency of detection of low-abundance transcripts in conditions of low background RNA. This might be a critical step when only small amounts of RNA are available and even more valuable when low-abundance transcripts should not be missed. Indeed, low-abundance transcripts are often important biological regulators (e.g. enhancers, silencers, transcription factors) and therefore should not be overlooked in a gene expression analysis. In such conditions, choosing an RT system such as SuperScript II that allows the detection of low-abundance transcripts would be adequate for global transcript profiling. In contrast, when qRT-PCR studies are performed in order to identify subtle differences, a representative profiling of the transcript abundance is expected. Indeed, a situation in which a gene present in low abundance in some samples is overestimated in comparison to samples where the transcript is present at higher levels is not suitable. It appears from our study that the RT inhibitor content largely influences the outcome profile of the PCR-detectable molecules. Therefore, this crucial step should be carefully set up when designing gene expression studies.

## Conclusion

While skills and experience are important for obtaining reliable results in qRT-PCR studies, identifying the source of variability is of prime importance. Indeed, there is no point in using an onerous and highly accurate molecular assay if the major sources of variability are underestimated. We observed that detection limits vary extensively among several commercial RT systems. When small differences among the RT systems (for a specific condition of total RNA amount) were measurable in the detection of an abundant transcript, the SuperScript II system was more efficient in conditions of low transcript abundance. However, it appears that the capacity for detecting low-abundance cDNA not only is attributable to the efficiency of the RT enzyme but is largely affected by some components in the RT system that bias the subsequent PCR amplification. Indeed, we quantified spurious differences among the RT systems when undiluted cDNA templates were used versus diluted samples. Therefore, a mandatory purification or dilution step (at least 10-fold) of cDNA templates will allow more accurate and sensitive measurements. In practice, any manufacturer's claims need to be treated cautiously. RT is an important step in a gene expression study and should be considered with infinite cautions when an absolute quantification is required.

## Methods

### Background RNA

Total RNA was extracted from 300 mg bovine testis tissue kept in RNA *later *(Ambion). Extraction followed a standard Trizol^® ^Reagent (Invitrogen) protocol. RNA was treated with bovine rDNase I (Ambion) and precipitated in isopropanol. The pellet was dissolved in nucleasefree water (Ambion) containing 1U/μl SUPERase•In™ (Ambion). The absence of genomic DNA was confirmed with a PCR design spanning an intron and using the bovine protamine 1 gene (*PRM1*; accession No. NW_930382) and the human deleted-in-azoospermia-like gene (*DAZL*; accession No. NM 001351), which are testis-specific gene sequences. RNA quantification was performed using a UV spectrophotometer (Ultrospec 3000, Pharmacia Biotech). The isolated RNA had an A_260_/A_280 _ratio of 2. Serial dilutions of testis RNA (1,000 ng/μl, 500 ng/μl, 50 ng/μl, 25 ng/μl, 12.5 ng/μl, and 5 ng/μl) were made in nuclease-free water containing 1 U/μl SUPERaseIn to perform the RT reactions.

### DNA standard curves

Reference genes, i.e. the enhanced green fluorescent gene (*EGFP*; accession no. U55762) and the oscillin gene (*GNPDA*; accession no. XM_881844), were amplified using primers specific to the desired application (standard curve, qRT-PCR, in vitro transcription, or end-point; [see Additional file 1]). Primers were designed using PrimerExpress 2.0 software (Applied Biosystems). Standard curves for the *EGFP *and *GNPDA *genes were constructed with a PCR product, using pEGFP-N1 plasmid (Clontech) or bovine testis cDNA, respectively, as template. PCR reaction was performed as follows: initial denaturation at 94°C for 2 min, followed by 35 cycles consisting of denaturation at 94°C for 30 s, annealing at 65°C for 30 s, and extension at 72°C for 45 seconds, with a final extension step at 72°C for 2 min. The single-band amplicons were visualised on a 1.5% agarose gel stained with ethidium bromide, purified using the QIAquick PCR Purification Kit (Qiagen), and verified by sequencing. For that purpose, the amplicons were cloned into the T/A plasmid vector pCRII (Invitrogen) according to the manufacturer's instructions. In order to confirm amplicon identities, sequencing reactions were performed using BigDye Terminator (version 3.1; Applied Biosystems) chemistry and a 9700 thermal cycler (Applied Biosystems). The PCR program for all sequencing reactions included initial denaturation at 96°C for 1 min, followed by 25 cycles consisting of denaturation at 96°C for 10 s, primer annealing at 50°C for 5 s, and extension at 60°C for 4 min. The sequencing products were purified by ethanol/EDTA precipitation, resuspended in a formamide solution (Applied Biosystems), and analysed with the ABI 3100-Avant capillary sequencer (Applied Biosystems). Gene homology was confirmed via the BLAST network service of the National Center for Biotechnology Information (NCBI) [31]. For each reference gene, the products of the PCR reactions were cleaned using a PCR purification kit (Qiagen). Dilutions of purified amplicons for qRT-PCR were made in nucleasefree water (Ambion).

### *EGFP *mRNA synthesis by in vitro transcription

Chimera primers containing a T7 promoter and a polyT tail [see Additional file 1] were used in a touchdown PCR in a GeneAmp 9700 (Applied Biosystems). PCR reaction was performed as follows: initial denaturation at 94°C for 3 min, followed by 10 cycles consisting of denaturation at 94°C for 30 s, annealing at 65°C for 30 s, and extension at 72°C for 45 s, with a reduction of the annealing temperature by 0.5°C during each cycle. PCR reaction was continued for an additional 33 cycles with annealing at 59°C for 30 s. PCR reaction was cleaned with the QIAquick PCR Purification Kit (Qiagen). T7 in vitro transcription using 275 ng *EGFP *PCR product was used to produce the spiking RNA to monitor RT with AmpliScribe T7, T3, and SP6 High Yield Transcription Kits (Epicentre). T7 transcription reaction was carried out for 2 h at 37°C, and the product was treated with DNase I. RNA was isolated with phenol/chloroform/isoamyl alcohol and precipitated with 7.5 M ammonium acetate, ethanol and linear acrylamide (Ambion). The pellet was washed and dissolved in nuclease-free water with 1U/μl SUPERaseIn, and the quantity of RNA was estimated with a 1:2,000 dilution of RiboGreen reagent (Molecular Probe).

### Reverse transcription

RNA samples used as background material for the RT assays were prepared (serial dilution from the same RNA pool; see above) in aliquots of 10, 25, 50,100, 1,000, and 2,000 ng total testis RNA. The aliquots were used once to avoid a freeze-and-thaw influence on the cDNA quality. All the RT reactions were prepared using one master mix for the respective enzyme, spiked with 1 fg or 1 pg *EGFP *mRNA template, and then distributed in the background RNA aliquots. Each RT assay was performed in a 20-μl reaction using oligo(dT)_12–18 _(Invitrogen) as primer and following the recommendations of the supplier. Unless otherwise stated, all the RT reactions were performed in triplicate, for each dilution point and each RT system, in a PTC-200 thermal cycler (MJ Research). All the RT systems screened in this study, i.e. Sensiscript and Omniscript (Qiagen), SuperScript™ II and SuperScript™ III (Invitrogen), and PowerScript™ (Clontech, Mountain View, CA), were used strictly in accordance with the recommendations of the respective suppliers.

### Detection of cDNA synthesis in RT reactions by real-time PCR

Quantitative RT-PCR was performed in triplicate in a 7700 SDS thermal cycler (Applied Biosystems). For quantification of *EGFP*, the hydrolysis probe technology (i.e. TaqMan probes [32]; [see Additional file 1]) was used as the detection system. The PCR mixture contained 12.5 μl 2X TaqMan Universal Master Mix (Applied Biosystems), 0.3 μM forward and reverse primers, 0.2 μM probe, and 2 μl cDNA (diluted or not). To reach the final volume of 25 μlper well, nuclease-free water was added. For quantification of *GNPDA*, the SYBR Green chemistry was used. Both the *EGFP *and the *GNPDA *genes were quantified using aliquots prepared from the same RT samples, avoiding variations attributed to the difference between RT assays. The absence of primer-dimers for both the *EGFP *and the *GNPDA *genes was validated using the SYBR Green I technique [33]. PCR reactions contained 12.5 μl 2X Power SYBR Green Master Mix (Applied Biosystems), 0.3 μM forward and reverse primers, and 0.25 U AmpErase UNG (Applied Biosystems). All runs (i.e. plates) included a triplicate of the standard curve and threeto six negative controls without target DNA that were partitioned at the beginning and the end of the plate. Thermal cycling conditions were as follows: 50°C for 2 min (incubation for the AmpErase UNG) preceding the first denaturation step at 95°C for 10 min, followed by 40 cycles of 95°C for 15 s and 60°C for 1 min. Melting curves analysis was performed for SYBR Green amplifications by plotting the fluorescence intensity in a graphic model. The presence of a single melting temperature peak (SYBR Green amplifications), which represented a specific amplicon, and that of the products of the TaqMan assays were confirmed by a run on a 2.5% agarose gel stained by SYBR gold nucleic acid stain (Invitrogen).

### Data analysis

Quantitative RT-PCR data were analysed using the appropriate threshold set up as recommended by Applied Biosystems [34] before being transferred to Excel. The slope of the calibration curve was calculated from the plot of base 10 log of initial target copy number versus corresponding Ct. The PCR efficiency (*E*) was determined from the slope of the curve obtained with serially diluted samples, as *E *= 10^(-1/slope)^, whereas the number of gene copies in each RT sample was calculated from the calibration curve. Basic statistical analyses were performed using Excel, and ANOVA was performed using SAS (Statistical Analysis System, Release 9.1, 2002, SAS Institute Inc., Cary, NC).

## Authors' contributions

JPLS and CT performed the preliminary molecular experiments. MD participated in the design and carried out the molecular studies. NB conceived the study, interpreted the results and wrote the manuscript. LD revised the manuscript. All authors read and approved the final manuscript.

## Supplementary Material

Additional file 1 Detailed values and parameters derived from standard curves obtained for the reference *EGFP *and *GNPDA *genes in the qRT-PCR assay. Results obtained from each point of the calibration curves used for the study. The calibration curves referred to the qPCR runs and were used to quantify the commercial RT systems: SensiScript, SuperScript II, SuperScript III, Omniscript and PowerScript. Click here for file

Additional file 2 Primers and probe sequences used in the study. A list of the primers and probe used in the study is presented. Whereas the primers for *PRM1 *were used to confirm the absence of genomic DNA in the testis RNA preparation, the primers of the *GNPDA *and *EGFP *were designed to establish the calibration curve or to perform the qRT-PCR assays. Click here for file

Additional file 3 Probabilities calculated on qRT-PCR data for the *EGFP *transcript quantified in undiluted RT samples. The statistics of the data presented in Figure 1 are reported. The probabilities (statistical analysis – SAS) are calculated on the real-time PCR measurements obtained for the *EGFP *transcript quantified in undiluted RT samples. Click here for file

Additional file 4 Probabilities calculated (statistical analysis – SAS) on qRT-PCR data for the *GNPDA *gene measured on undiluted RT samples. The statistics of the data presented in Table 2 and 3 are reported. The probabilities (statistical analysis – SAS) are calculated on the real-time PCR measurements obtained for the *GNPDA *transcript quantified in undiluted RT samples performed with the 5 commercial RT systems. Click here for file
