# Supplementary material for: Detection limits of several commercial reverse transcriptase enzymes: impact on the low- and high-abundance transcript levels assessed by quantitative RT-PCR
Source: BMC Mol Biol. 2007 Oct 22;8:93. doi: 10.1186/1471-2199-8-93 (PMC2151766; doi:10.1186/1471-2199-8-93)
Supplement: Additional file 1 — Detailed values and parameters derived from standard curves obtained for the reference EGFP and GNPDA genes in the qRT-PCR assay. Results obtained from each point of the calibration curves used for the study. The calibration curves referred to the qPCR runs and were used to quantify the commercial RT systems: SensiScript, SuperScript II, SuperScript III, Omniscript and PowerScript. [file 1471-2199-8-93-S1.doc]

**Additional Table 1a**. Detailed Ct values derived from standard curves^a^ obtained for the reference EGFP gene in the TaqMan assay.

| RT systems^a^ |  | Ct value mean^b^ | | | | | | | | r^2^ | Eff.^c^ |
| --- | --- | --- | --- | --- | --- | --- | --- | --- | --- | --- | --- |
|  | EGFP^d^ | NTC | 130 | 649 | 1298 | 32452 | 129810 | 259620 | NTC |  | % |
| SensiS | 1 fg | 40  (0) | 33.45 (1.34) | 30.49 (0.54) | 29.78  (1.61) | 25.17 (0.98) | 22.27 (0.62) | 21.35  (0.82) | 40  (0) | 0,9933 | 89.3 |
|  | 1 pg | 38.86  (3.57) | 34.04  (1.14) | 30.80  (0.64) | 29.98  (0.70) | 25.91  (0.75) | 23.09  (0.55) | 21.95  (0.80) | 40  (0) | 0.9911 | 93.1 |
| SS II | 1 fg | 39.44  (2.03) | 34.12  (0.31) | 30.41  (0.67) | 28.92  (0.66) | 24.93  (1.10) | 22.34  (0.09) | 21.41  (0.15) | 36.61  (1.72) | 0.9872 | 89.7 |
|  | 1 pg | 37.70  (5.36) | 31.97  (0.33) | 29.37  (0.22) | 28.17  (0.40) | 23.64  (0.50) | 21.39  (0.19) | 20.64  (0.25) | 37.59  (8.11) | 0.9986 | 95.8 |
| SS III | 1 fg | 40  (0) | 32.22  (0.51) | 30.17  (0.06) | 28.87  (0.52) | 24.08  (0.68) | 22.53  (0.27) | 20.92  (0.54) | 40  (0) | 0.9969 | 97.6 |
|  | 1 pg | 39.38  (2.74) | 32.26  (0.12) | 30.77  (0.53) | 29.43  (0.14) | 24.22  (0.29) | 23.05  (0.20) | 21.00  (0.51) | 39.94  (0.27) | 0.9899 | 96.5 |
| OmniS | 1 fg | - | - | - | - | - | - | - | - | - | - |
|  | 1 pg | 40  (0) | 32.69  (2.17) | 30.16  (0.56) | 28.94  (1.31) | 24.22  (0.29) | 22.13  (0.28) | 20.84  (0.28) | 40  (0) | 0.9954 | 91.8 |
| PowerS | 1 fg | 39.50  (2.21) | 32.59 (0.17) | 29.83 (0.72) | 28,75 (0.39) | 23.91 (0.11) | 21.88 (0.35) | 20.83 (0.50) | 40  (0) | 0.9989 | 92.21 |
|  | 1 pg | - | 33.05  (0.41) | 32.36  (0.15) | 30.64  (0.42) | 25.27  (0.18) | 23.93  (N/A) | 22.24  (0.16) | 40  (0) | 0.9847 | 93.5 |

^a^The standard curves referred to the qPCR runs and were used to quantify the RT systems: SensiScript (SensiS), SuperScript II (SS II), SuperScript III (SS III), Omniscript (OmniS) and PowerScript (PowerS).

^b^Coefficients of variation (%) are given in parentheses.

^c^Amplification kinetics for each calibration curve within each plate.

^d^The number of molecules was calculated based on an average quantity measured by spectrophotometry (ABS 260/280) converted in number of molecules using the following formula: M.W. of dsDNA = (number of nucleotides in the DNA fragment × 607.4) + 157.9. NTC, No Template Control.

**Additional Table 1b**. Compilation of Ct values derived from the standard curves^a^ obtained for the reference GNPDA gene.

| RT systems^a^ | Ct value mean^b^ | | | | | | | R^2^ | E^c^ |
| --- | --- | --- | --- | --- | --- | --- | --- | --- | --- |
| *No. mol*.^d^ | *NTC* | *17* | *33* | *332* | *1660* | *3320* | *NTC* |  | % |
| SensiS | 40  (0) | - | 33.17  (1.31) | 29.53  (0.72) | 26.58  (0.19) | 25.71  (0.34) | 40  (0) | 0.9936 | 83.7 |
| SuperSII | 39.71  (1.26) | 34.05  (0.51) | 32.26  (0.46) | 28.16  (0.20) | 25.49  (0.77) | 24.60  (0.11) | 39.03  (1.15) | 0.9946 | 74.6 |
| SuperSIII | 40  (0) | 33.97  (0.08) | 32.95  (0.90) | 28.56  (0.85) | 26.17  (0.52) | 25.27  (0.29) | 40  (0) | 0.9943 | 81.6 |
| OmniS | 40  (0) | 34.55  (1.25) | 33.42  (1.54) | 29.16  (0.40) | 26.52  (0.42) | 25.66  (0.22) | 40  (0) | 0.9932 | 79.4 |
| PowerS | 40  (0) | 33.31  (1.93) | 32.21  (0.85) | 29.33  (0.50) | 26.47  (0.23) | 25.40  (0.17) | *22.28*^†^  *(0.17)* | 0.9947 | 97.9 |

^a^The standard curves referred to the qPCR runs and were used to quantify the RT systems : SensiScript (SensiS), SuperScript II (SuperSII), SuperScript III (SuperSIII), Omniscript (OmniS) and PowerScript (PowerS).

^b^Coefficients of variation (%) are given in parenthesis.

^c^Amplification kinetics for each calibration curve within each plate.

^d^The number of molecules was calculated based on an average quantity measured by spectrophotometry (ABS 260/280) converted to number of molecules using the following formula: M.W. of dsDNA = (number of nucleotides in the DNA fragment × 607.4) + 157.9.

^†^ Not a NTC (No Template Control) value but a dilution point at 33202 copies measured in triplicate.

Coefficient of correlation (*R^2^*) and amplification efficiency (*E*) are derived from calibration curve; standard deviation, in parenthesis.
