# Supplementary material for: Detection limits of several commercial reverse transcriptase enzymes: impact on the low- and high-abundance transcript levels assessed by quantitative RT-PCR
Source: BMC Mol Biol. 2007 Oct 22;8:93. doi: 10.1186/1471-2199-8-93 (PMC2151766; doi:10.1186/1471-2199-8-93)
Supplement: Additional file 2 — Primers and probe sequences used in the study. A list of the primers and probe used in the study is presented. Whereas the primers for PRM1 were used to confirm the absence of genomic DNA in the testis RNA preparation, the primers of the GNPDA and EGFP were designed to establish the calibration curve or to perform the qRT-PCR assays. [file 1471-2199-8-93-S2.doc]

**Additional Table 2.** Primers and probe sequences ^a^.

| **Gene**^b^ | **PCR type** |  | **Sequence (5’-3’)** | **Product size (bp)** |
| --- | --- | --- | --- | --- |
| *EGFP* | Standard curve | Forward | TAATACGACTCACTATAGGATGGTGAGCAAGGGCGAG | 732 |
|  |  | Reverse | TTTTTTTTTTTTTTTTTTTTTTTTTTTTTTGCGGCGGTCACGAACTC |  |
|  | qRT-PCR (TaqMan) | Forward | CTGCTGCCCGACAACCAC | 73 |
|  |  | Reverse | TGTGATCGCGCTTCTCGTT |  |
|  |  | Probe ^c^ | *FAM*-ACCTGAGCACCCAGTCCGCCCT-*TAMRA* |  |
|  | End-point | Forward | GCATCGACTTCAAGGAGGAC | 290 |
|  |  | Reverse | GAACTCCAGCAGGACCATGT |  |
|  | T7 transcription | Forward | TAATACGACTCACTATAGGATGGTGAGCAAGGGCGAG | 732 |
|  |  | Reverse | TTTTTTTTTTTTTTTTTTTTTTTTTTTTTTGCGGCGGTCACGAACTC |  |
| *GNDPA* | Standard curve | Forward | AGGTGCTCACAAGGCGTTTG | 287 |
|  |  | Reverse | AGGTACTTGATACCAGGATTAGCACAG |  |
|  | qRT-PCR (SYBR Green I) | Forward | CCATCGAGGAAGGAGTGAACC | 78 |
|  |  | Reverse | CGTCGCACACAAACACTGTG |  |
|  | End-point | Forward | AGGTGCTCACAAGGCGTTTG | 287 |
|  |  | Reverse | 5’-AGGTACTTGATACCAGGATTAGCACAG |  |
| *PRM1*^d^ | End-point | Forward | AGATACCGATGCTGCCTCAC | 234 |
|  |  | Reverse | GTGGCATGTTCAAGATGTGG |  |

^a^Primers and probe were synthesised by Applied Biosystems.

^b^*EGFP*: Enhanced Green Fluorescent Protein (GenBank accession no. U55762); *GNPDA*: Glucosamine-6-phosphate isomerase (GenBank accession no. XM_881844); *PRM1*: protamine 1 (GenBank accession no. NW_930382).

^c^Characteristics of the probe used in the TaqMan assay: FAM: 6-carboxyfluorescein; TAMRA: carboxytetramethylrhodamine.

^d^Primers used to test for absence of genomic DNA
