# Supplementary material for: Detection limits of several commercial reverse transcriptase enzymes: impact on the low- and high-abundance transcript levels assessed by quantitative RT-PCR
Source: BMC Mol Biol. 2007 Oct 22;8:93. doi: 10.1186/1471-2199-8-93 (PMC2151766; doi:10.1186/1471-2199-8-93)
Supplement: Additional file 3 — Probabilities calculated on qRT-PCR data for the EGFP transcript quantified in undiluted RT samples. The statistics of the data presented in Figure 1 are reported. The probabilities (statistical analysis – SAS) are calculated on the real-time PCR measurements obtained for the EGFP transcript quantified in undiluted RT samples. [file 1471-2199-8-93-S3.doc]

**Additional Table** **3.** Probabilities calculated on qRT-PCR data for the *EGFP* transcript quantified in undiluted RT samples^a^

| **EGFP**  **(fg)** | **Bkg RNA**  **(ng)** | **PowerScript**  **Vs**  **SensiScript** | **PowerScript**  **Vs**  **SuperScriptII** | **PowerScript**  **vs**  **SuperScriptIII** | **SensiScript**  **Vs**  **SuperScriptII** | **SensiScript**  **Vs**  **SuperScriptIII** | **SuperScriptII**  **Vs**  **SuperScriptIII** | **OmniScript**^b^  **Vs**  **PowerScript** | **OmniScript**^b^  **Vs**  **SensiScript** | **OmniScript**^b^  **Vs**  **SuperScriptII** | **OmniScript**^b^  **Vs**  **SuperScriptIII** |
| --- | --- | --- | --- | --- | --- | --- | --- | --- | --- | --- | --- |
| 1 | 0 | 0.9556 | <.0001 | 0.1763 | <.0001 | 0.1306 | <.0001 | . | . | . | . |
| 1 | 10 | 0.9816 | 0.0009 | 0.9866 | 0.0006 | 0.8945 | 0.0013 | . | . | . | . |
| 1 | 25 | 0.9239 | <.0001 | 0.9492 | <.0001 | 0.6733 | <.0001 | . | . | . | . |
| 1 | 50 | 0.5476 | 0.0002 | 0.9989 | 0.0009 | 0.4736 | 0.0002 | . | . | . | . |
| 1 | 100 | 0.0595 | <.0001 | 0.9997 | <.0001 | 0.0521 | <.0001 | . | . | . | . |
| 1 | 1000 | 0.9653 | <.0001 | 0.9892 | <.0001 | 0.8660 | <.0001 | . | . | . | . |
| 1 | 2000 | 0.9990 | 0.0039 | 0.9949 | 0.0047 | 0.9996 | 0.0053 | . | . | . | . |
| 1000 | 0 | 0.0564 | 0.1918 | 0.0944 | 0.0016 | 0.9968 | 0.0026 | 0.0022 | 0.2622 | 0.0001 | 0.1633 |
| 1000 | 10 | 0.0001 | 0.0013 | 0.0254 | 0.3260 | <.0001 | <.0001 | <.0001 | <.0001 | <.0001 | 0.0127 |
| 1000 | 25 | 0.8736 | 0.7577 | 0.1370 | 0.2790 | 0.0322 | 0.6166 | 0.0194 | 0.0047 | 0.1211 | 0.7133 |
| 1000 | 50 | 0.0111 | 1.0000 | 0.8409 | 0.0117 | 0.0025 | 0.8243 | 0.0217 | <.0001 | 0.0206 | 0.1050 |
| 1000 | 100 | 0.0525 | 1.0000 | 0.3177 | 0.0532 | 0.0025 | 0.3141 | 0.0002 | <.0001 | 0.0002 | 0.0033 |
| 1000 | 1000 | 0.6703 | 0.0036 | 0.0179 | 0.0270 | 0.1420 | 0.8124 | 0.0367 | 0.2754 | 0.5584 | 0.9888 |
| 1000 | 2000 | 0.5134 | 0.0147 | 0.0078 | 0.1779 | 0.0949 | 0.9924 | 0.0239 | 0.2764 | 0.9975 | 0.9428 |

^a^Statistics of the data presented in Figure 1; yellow boxes represent a significant difference (*P* < 0.05), turquoise boxes suggest a trend (0.05 < *P* < 0.1)

No data available from the OmniScript assay performed with low transcripts amount (1 fg of *EGFP*) since no signal was detected
