# Supplementary material for: Detection limits of several commercial reverse transcriptase enzymes: impact on the low- and high-abundance transcript levels assessed by quantitative RT-PCR
Source: BMC Mol Biol. 2007 Oct 22;8:93. doi: 10.1186/1471-2199-8-93 (PMC2151766; doi:10.1186/1471-2199-8-93)
Supplement: Additional file 4 — Probabilities calculated (statistical analysis – SAS) on qRT-PCR data for the GNPDA gene measured on undiluted RT samples. The statistics of the data presented in Table 2 and 3 are reported. The probabilities (statistical analysis – SAS) are calculated on the real-time PCR measurements obtained for the GNPDA transcript quantified in undiluted RT samples performed with the 5 commercial RT systems. [file 1471-2199-8-93-S4.doc]

**Additional Table 4.** Probabilities calculated (statistical analysis – SAS) on qRT-PCR data for the *GNPDA* gene measured on undiluted RT samples^a^.

| **Bkg RNA**  **(ng)** | **PowerScript**  **Vs**  **SensiScript** | **PowerScript**  **Vs**  **SuperScriptII** | **PowerScript**  **vs**  **SuperScriptIII** | **SensiScript**  **Vs**  **SuperScriptII** | **SensiScript**  **Vs**  **SuperScriptIII** | **SuperScriptII**  **Vs**  **SuperScriptIII** |
| --- | --- | --- | --- | --- | --- | --- |
| 0 | 0.0083 | 0.0083 | 0.0083 | 1.0000 | 1.0000 | 1.0000 |
| 10 | 0.3022 | 0.0511 | 0.9959 | 0.5914 | 0.2272 | 0.0375 |
| 25 | 0.8451 | 0.2089 | 0.8448 | 0.5540 | 0.4136 | 0.0670 |
| 50 | 0.1512 | 0.0190 | 0.9831 | 0.4272 | 0.3291 | 0.0510 |
| 100 | 0.0072 | 0.0036 | 0.9803 | 0.9417 | 0.0118 | 0.0058 |
| 1000 | <.0001 | <.0001 | 0.9554 | 0.0073 | <.0001 | <.0001 |
| 2000 | <.0001 | 0.0019 | 0.9918 | 0.0006 | <.0001 | 0.0014 |

^a^Yellow boxes represent significant difference *P* < 0.05; turquoise boxes suggest a trend (0.05 > *P* < 0.1)
